# Examining the Relationships Among Concealment Tendencies, Illness Attitudes, Belief in a Just World, and Cognitive Flexibility

**DOI:** 10.3389/fpsyg.2021.627739

**Published:** 2021-09-03

**Authors:** Kyung Hwan Cha, Hua Jin, Jung Hee Ha, Juliet Jue

**Affiliations:** ^1^Department of Multicultural Education, Hanyang University, Seoul, South Korea; ^2^Graduate School of Counseling Psychology, Hanyang University, Seoul, South Korea; ^3^Department of Art Therapy, Hanyang Cyber University, Seoul, South Korea

**Keywords:** concealment tendencies, illness attitudes, belief in a just world, cognitive flexibility, control, alternatives

## Abstract

The purpose of this study was to verify the relationships among concealment tendencies, illness attitudes, belief in a just world, and cognitive flexibility. The participants were 418 Korean and 400 Chinese adults. We conducted correlational analysis, structural equation modeling, and verification of mediating effects. We found that cognitive flexibility–control factor fully mediated the relationship between concealment tendencies and illness attitudes for Korean participants and partially mediated the relationship for Chinese participants. The relationship between concealment tendencies and cognitive flexibility–alternatives factor differed across participants’ country of origin. For Chinese participants, cognitive flexibility–alternatives fully mediated the relationship between concealment tendencies and belief in a just world. These differences might stem from the countries’ different social systems, values, and attitudes. Finally, we discuss this study’s implications and limitations.

## Introduction

Asian cultures strongly value groups and communities, and Asian people are known to be highly sensitive to others’ judgments. For example, Koreans feel more embarrassed when they make mistakes in front of others than while alone, and they try to hide these public mistakes (Lee and Kim, 2006). These cultural characteristics are common not only for Koreans but also for Chinese. In collectivist cultures, unity and harmony with others are of great importance (Wang, 2011). Collectivist cultures allow people to rely on and cooperate with each other; however, they may overemphasize interpersonal relationships, making people hyperconscious of what others think or appreciate. People in collectivist cultures fear public mistakes, change or hide their opinions based on others’ evaluations, and strive to maintain perfect images of themselves (Wang, 2011). Thus, concealing mistakes or flaws could serve to protect one’s perceived perfection.

*Concealment tendencies* are related to perfectionistic self-presentation, which is defined as the desire to appear perfect to others (Hewitt et al., 2003). People with such tendencies tend to show only their perfect qualities and hide imperfect behavior or language. Sub-factors of perfectionistic self-presentation include perfectionistic self-promotion (PSP), non-display of imperfection (NDP), and non-disclosure of imperfection (NDC). Concealment tendencies comprise the latter two categories.

Concealment tendencies are associated with psychological and behavioral maladjustment (Kim and Seo, 2009; Hong and Hong, 2011; Kim and Hyun, 2012; Kim and Lee, 2013; Park and Yang, 2014). Previous studies have shown that the characteristics of perfectionism dominated by concealment tendencies include fear of rejection (Wu and Wei, 2008; Flett et al., 2014), negativity bias (Kim and Hyun, 2012), maladaptive emotions (Go and Hyeon, 2009), and selective disclosure of imperfection (Hewitt et al., 2003; Lee and Suh, 2010; Baek and Lee, 2013). In contrast, Li et al. (2006b) reported on the positive functions of concealment tendencies. The researchers argue that concealment tendencies give people a sense of psychological stability by promoting a positive self-image, avoiding the hatred of others, and preventing harm to the individual’s reputation. Further, short-term self-concealment is advantageous for perfectionists to avoid the evaluation of others, so this can also be viewed as an adaptive behavior. Additionally, research has determined that adolescents’ concealment of themselves from their parents may be beneficial for the development of emotional autonomy (Li et al., 2006a).

Nonetheless, with the global spread of coronavirus disease (COVID-19) in 2020, individuals with strong concealment tendencies are more likely to experience anxiety and health concerns due to their personality traits. As pointed out by Lu et al. (2009), those with strong concealment tendencies also are more likely to develop stress-related illnesses. The spread of infectious disease augments people’s health concerns. Kim et al. (2020) conducted a survey of 1,000 Koreans and found that, compared with natural disasters or non-contagious diseases, COVID-19 has induced higher levels of stress and anxiety. Similarly, Chinese citizens have also reported feeling high levels of anxiety and fear from the spread of COVID-19 (Li et al., 2020).

Additionally, the issue of quarantine beyond the individual level has become important. When their anxiety increases, people may lose faith in society or develop negative attitudes. Yang and Wang (2020) reported that the spread of COVID-19 has undermined college students’ confidence in the government and has created tensions between the government and citizens, threatening stability. On the contrary, Li et al. (2020) surveyed citizens both within and outside Hubei, the first COVID-19 outbreak area, and reported that their satisfaction with the government’s countermeasures was high (85.7 and 90.0%, respectively). Kim et al. (2020) conducted a similar survey on metropolitan area residents in Korea and reported that 63.3% of study participants were satisfied with the government’s countermeasures. One of the reasons why people evaluate the government’s response differently might be that individuals have different levels of security or belief in their society and country. In this study, we examined whether Korean and Chinese citizens feel secure in their respective countries through *belief in a just world*.

Belief in a just world is defined as personal assumptions that the world is basically fair (Lerner, 1977). These beliefs provide psychological stability by recognizing the physical and social environment to which an individual belongs as orderly and stable (Lerner, 1977; Dalbert, 1999; Furnham, 2003). There may be unavoidable inequality in various areas (e.g., wealth, educational opportunities, and welfare), but responses to the unfairness vary from person to person (Nudelman and Shiloh, 2011). The belief that everything in the world happens fairly creates hope, trust, and confidence despite an uncertain future (Du et al., 2007; Zhen, 2013). Previous research has demonstrated that perceptions of unfairness cause psychological maladjustment, such as depression and anxiety, and *cognitive flexibility* mediates this process (Kim et al., 2012; Choi and Shin, 2018; Shin, 2018).

Cognitive flexibility refers to the ability to perceive that one can control difficult situations and produce a variety of alternatives to the problem at hand (Dennis and Vander Wal, 2010). It is the ability to change (Thurston and Runco, 1999), the spontaneity to adapt to situations, recognizing that there are new alternatives and choices an individual can make (Martin and Rubin, 1994; Heo, 2011), or personal ability to cope with unexpected changes (Gough, 1995). Thus, a measure of cognitive flexibility is the degree to which an individual challenges negative thinking and restructures the situations when faced with difficulties (Cho and Jo, 2017).

Cognitive flexibility is divided into two sub-factors: alternatives and control. While the former involves self-efficacy with respect to presenting alternative interpretations of and solutions to difficult situations, the latter is defined as the tendency to perceive the situation as controllable (Dennis and Vander Wal, 2010). According to Ellis (2001), rigid thinking, including irrational thoughts or self-defeating beliefs, causes cognitive distortion and increases anxiety. In contrast, cognitive flexibility promotes positive problem solving and lowers emotional distress, including anxiety (Johnco et al., 2013; Kim and You, 2017; Shin, 2018). Wang et al. (2019) found that cognitive flexibility (both dimensions) predicted belief in a just world. Heo (2011) reports that cognitive flexibility–control exhibited strong negative correlations with dysfunctional perfectionism, depression, and anxiety. The researcher also found that cognitive flexibility–control partially mediated the relationship between dysfunctional perfectionism and depression as well as the relationship between dysfunctional perfectionism and anxiety. Therefore, we assume that cognitive flexibility mediates the process of concealment tendencies affecting illness attitudes and belief in a just world.

**Figure 1:**
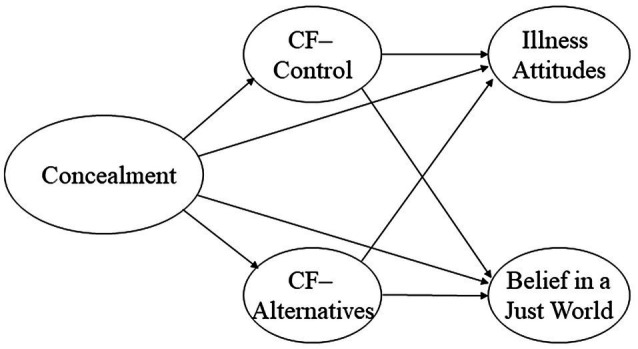
Research Model.

Based on the preceding studies, we hypothesized that the relationships among concealment tendencies, illness attitudes, belief in a just world, and cognitive flexibility would be as they are presented in Figure 1. In an effort to determine the existence of cultural differences, we examined the relationships among the foregoing variables for both Korean and Chinese participants.

## Materials and Methods

### Participants and Procedures

We surveyed a total of 818 adults in the Republic of Korea and China. In the Korean subsample, there were 145 men (34.7%) and 273 women (65.3%). Their age distribution was as follows: 106 in their 20s (25.4%), 122 in their 30s (29.2%), 94 in their 40s (22.5%), 94 in their 50s (22.5%), and 2 in their 60s or older (0.5%). One hundred and ninety-four were married (46.4%), 212 were single/divorced/widowed (50.7%), and 12 specified “other” or provided no answer to the marital status item (2.9%). With respect to past disease experiences, 105 participants (25.1%) had diseases, 311 (74.4%) had no serious disease, and two specified “other” or provided no answer to the item (0.5%). Regarding current disease experiences, 71 participants (17.0%) have diseases, while 347 (83.0%) have no serious disease. As for educational background, 86.2% were college graduates, and 13.6% were high school graduates.

In the Chinese subsample, there were 181 men (45.3%) and 219 women (54.7%). Their age distribution was as follows: 215 in their 20s (53.6%), 75 in their 30s (18.8%), 40 in their 40s (10.0%), 67 in their 50s (16.8%), and 3 in their 60s and over (0.8%). Two hundred and sixteen were married (54.0%), 181 were single/divorced/widowed (45.2%), and three specified “other” or provided no answer to this item (0.8%). With respect to past disease experiences, 32 participants (0.8%) had diseases, while 368 (92.0%) had no serious disease. Regarding current disease experiences, 31 participants (7.8%) have diseases, while 369 (92.2%) have no serious disease. As for educational background, 78.7% were college graduates, and 15.5% were high school graduates.

The survey period lasted from April 2020 to May 2020. We used both paper and online surveys. After excluding incomplete paper surveys and online surveys completed too rapidly (less than 100 s), 418 Korean questionnaires and 400 Chinese questionnaires remained for the final analysis.

### Measures

#### Perfectionistic Self-Presentation Scale

Hewitt et al. (2003) developed the Perfectionistic Self-Presentation Scale (PSPS). We used the PSPS–Korean Version translated and modified by Ha (2011) to measure Korean participants’ concealment tendencies. The scale comprises 19 items. In this study, we used 11 items from the scale representing two domains: (1) NDP and (2) NDC. Items were rated on a 7-point Likert scale ranging from 1 (*not at all likely*) to 7 (*extremely likely*). Higher total scores represent higher levels of perfectionistic self-presentation. Ha reported a Cronbach’s *α* of 0.85 for the full scale. In this study, we found that Cronbach’s *α* to be 0.76 for all items, 0.67 for NDP, and 0.66 for NDC.

For Chinese participants, we used the PSPS–Chinese Version translated and validated by Yu (2007). This scale comprises 21 items, and we used 12 items from the scale representing the same two domains as above. Its rating scale and interpretation of total scores match those of the PSPS–Korean Version. Yu reported a Cronbach’s *α* of 0.86 for the full scale. In this study, we found that Cronbach’s *α* to be 0.82 for all items, 0.74 for NDP, and 0.77 for NDC.

#### Cognitive Flexibility Inventory

To measure cognitive flexibility, we used the Cognitive Flexibility Inventory (CFI) developed by Dennis and Vander Wal (2010). For Korean participants, we used the CFI–Korean Version translated and modified by Heo (2011). This inventory comprises 19 items representing two domains: control (eight items) and alternatives (11 items). The cognitive flexibility–control subscale measures the tendency to perceive difficult situations as controllable. For example, respondents answer statements, such as When I face a difficult situation, I feel like I will lose control; When I face a difficult situation, I get so stressed that I can’t think of any way to deal with it.

On the other hand, the cognitive flexibility–alternatives subscale measures the ability to conceive various alternative explanations for life events and human behaviors and produce alternative solutions to difficult situations. For example, this scale presents statements, such as When faced with difficult situations, I consider various options before acting; I tend to view difficult situations from different points of view. These two subscales measure different constructs: The cognitive flexibility–alternative factor measures the consideration of various solutions. The cognitive flexibility–control subscale measures the sense of efficacy based on the belief in flexibility (Johnco et al., 2014).

Items were rated on a 5-point Likert scale ranging from 1 (*not at all likely*) to 5 (*extremely likely*). Higher total scores represent higher levels of cognitive flexibility. Heo (2011) reported high internal consistency for the full scale (*α* = 0.86). In this study, we found that Cronbach’s *α* to be 0.90 for all items, 0.84 for Cognitive Flexibility–Control, and 0.90 for Cognitive Flexibility–Alternative.

For Chinese participants, we used the CFI–Chinese Version translated and validated by Wang et al. (2016). The scale comprises 20 items, including 8 items for control and 12 items for alternatives. The characteristics of the scale match those of the CFI–Korean Version. Wang et al. reported the Cronbach’s *α* for the scale as 0.88. We found that Cronbach’s *α* to be 0.88 for all items, 0.79 for Cognitive Flexibility–Control, and 0.92 for Cognitive Flexibility–Alternative.

#### Illness Attitudes Scale

To measure illness attitudes, we used the Illness Attitudes Scale (IAS) developed by Kellner (1987). For Korean participants, we used the IAS–Korean Version translated and validated by Yi (2004). This scale consists of 27 items spanning eight factors: (1) fear of illness, (2) treatment experiences, (3) health habits, (4) effects of symptoms, (5) fear of disease, (6) disease conviction, (7) obsession with somatic sensations, and (8) fear of death. Items were rated on a 5-point Likert scale ranging from 1 (*not at all likely*) to 5 (*extremely likely*). Higher total scores reflect more negative illness attitudes. The scale demonstrated high internal consistency in Yi’s study (*α* = 0.86). We found Cronbach’s *α* to be 0.84 for the total scale.

For Chinese participants, we used the IAS–Chinese Version produced by Luo et al. (2013). The scale comprises 20 items representing four domains: (1) fear of illness and death, (2) effects of symptoms, (3) behaviors of prevention and treatment of disease, and (4) doubts about one’s own health. Its rating scale and interpretation of total scores match those of the IAS–Korean Version. Luo et al. reported the subscales’ reliability coefficients as 0.82, 0.82, 0.74, and 0.68, respectively. We found Cronbach’s *α* to be 0.80 for the total scale.

As described above, we used the IAS developed by Kellner (1987) in Korea and China; the scale used by the two countries is the same. Researchers validated the IAS in Korea and China and found results related to depression and anxiety in both countries (Kim, 2005; Yi, 2004; Luo et al., 2013). However, its sub-factors were different for each country, with eight factors for Korea and four factors for China. Thus, we randomly allocated items to three parcels, which were the same for the two countries’ samples, and used them for analysis.

#### Belief in a Just World Scale

To measure belief in a just world, we employed the Belief in a Just World Scale (BJW) developed by Dalbert (1999). For Korean participants, we used the BJW–Korean Version translated and revised by Jung and Ahn (2017). It has two subscales: (1) BJW—self and (2) BJW—others. The scale consists of 13 items, each rated on a 6-point Likert scale ranging from 1 (*not at all likely*) to 6 (*extremely likely*). Higher total scores indicate stronger belief in a just world. Jung and Ahn reported high internal consistency for the full scale in their study (*α* = 0.88). We found that Cronbach’s *α* for all items was 0.85. For sub-factors, Cronbach’s *α* for personal belief was 0.78, and Cronbach *α* for general belief was 0.78.

For Chinese participants, we used the BJW–Chinese Version translated and revised by Su et al. (2012). The characteristics of the scale match those of the BJW–Korean Version. Su et al. reported high internal consistency for the full scale (*α* = 0.89). In this study, we found that Cronbach’s *α* to be 0.94 for all items, 0.93 for personal belief, and 0.88 for general belief sub-scale.

### Analysis Method

We used AMOS 20 and IBM’s SPSS Statistics 24.0 to test study hypotheses through structural equation modeling. We calculated the reliability for each measured variable in addition to calculating descriptive statistics and conducting correlational analysis. We conducted a confirmatory factor analysis to verify the validity of the measurement model and used a structural equation to verify the validity of the theoretical model and the influences of the variables. Finally, we used phantom variables to verify the statistical significance and relative size of the mediating effect.

## Results

### Correlations Among Variables

First, we conducted item parceling as suggested by Russell et al. (1998). Then, we calculated means and standard deviations (see Table 1) and computed correlations among the measured variables in both subsamples (see Table 2).

**Table 1:** Descriptive statistics for measurement variables.

	Variable	Mean	SD	Skewness	Kurtosis
Korean subsample (*N* = 418)	Concealment	4.53	0.83	−0.11	−0.04
	Cognitive flexibility–control	3.43	0.75	−0.05	−0.46
	Cognitive flexibility–alternatives	3.92	0.62	−0.36	−0.11
	Illness attitudes	2.67	0.49	0.02	−0.48
	Belief in a just world	3.60	0.73	−0.19	0.59
Chinese subsample (*N* = 400)	Concealment	4.39	0.97	−0.20	−0.13
	Cognitive flexibility–control	3.25	0.57	0.37	−0.03
	Cognitive flexibility–alternatives	3.51	0.64	−0.47	1.22
	Illness attitudes	2.61	0.64	0.36	−0.08
	Belief in a just world	4.05	0.98	−0.18	−0.01

**Table 2:** Correlation coefficients for measurement variables.

S. No.	Variables	1	2	3	4	5
1	Concealment	–	−0.27^**^	0.30^**^	0.36^**^	0.19^**^
2	Cognitive flexibility–control	−0.17^**^	–	0.19^**^	−0.43^**^	0.09
3	Cognitive flexibility–alternatives	0.05	0.42^**^	–	0.11^*^	0.39^**^
4	Illness attitudes	0.18^**^	−0.27^**^	−0.08	–	0.12^*^
5	Belief in a just world	−0.09	0.28^**^	0.22^**^	0.00	–

*Correlations upper half: Chinese subsample; correlations lower half: Korean subsample*.

* *p < 0.05;*

** *p < 0.01*.

### Verification of Measurement Models

We conducted confirmatory factor analysis to verify that the measured variables accurately reflected the latent variables. In the Korean subsample, the results of the confirmatory factor analysis were as follows: *x*^2^ (55, *N* = 418) = 177.62, *p* < 0.001, TLI = 0.91, CFI = 0.93, and RMSEA = 0.07 (0.06~0.09). As for the Chinese subsample, the confirmatory factor analysis produced the following results: *x*^2^ (55, *N* = 400) = 221.29, *p* < 0.001, TLI = 0.91, CFI = 0.93, and RMSEA = 0.09 (0.08~0.10). Thus, we judged the measurement models in this study to be suitable.

### Verification of Structural Models

In the Korean subsample, we compared the research model and the modified model to determine the one that most adequately represents the structural relationships among the variables (see Table 3). In the modified model, there is neither a path from concealment tendencies to illness attitudes nor a path from concealment tendencies to belief in a just world. We conducted a chi-square test for comparison because the research model and the modified model are mutually nested models. In order for the modified model to replace the research model, the chi-square value needed to be 3.84 or greater when the degrees of freedom of the difference equaled 1 and the significance level was set to 0.05. The research model had a chi-square value of 177.62, while the modified model’s corresponding value was 180.48. When the degrees of freedom of the difference equaled 2, the chi-square value was 2.86, representing a significant change (*p* < 0.001). Therefore, due to its superior fit, we selected the modified model as the final model.

**Table 3:** Fit of research model and of modified model.

	Model	*χ* ^2^	*df*	TLI	CFI	RMSEA
Korean subsample (*N* = 418)	Research model	177.62^***^	55	0.91	0.93	0.07 (0.06–0.09)
	Modified model	180.48^***^	57	0.91	0.93	0.07 (0.06–0.08)
Chinese subsample (*N* = 400)	Research model	221.29^***^	55	0.91	0.93	0.09 (0.08–0.10)
	Modified model	227.45^***^	58	0.91	0.93	0.09 (0.07–0.10)

*** *p < 0.001*.

**Figure 2:**
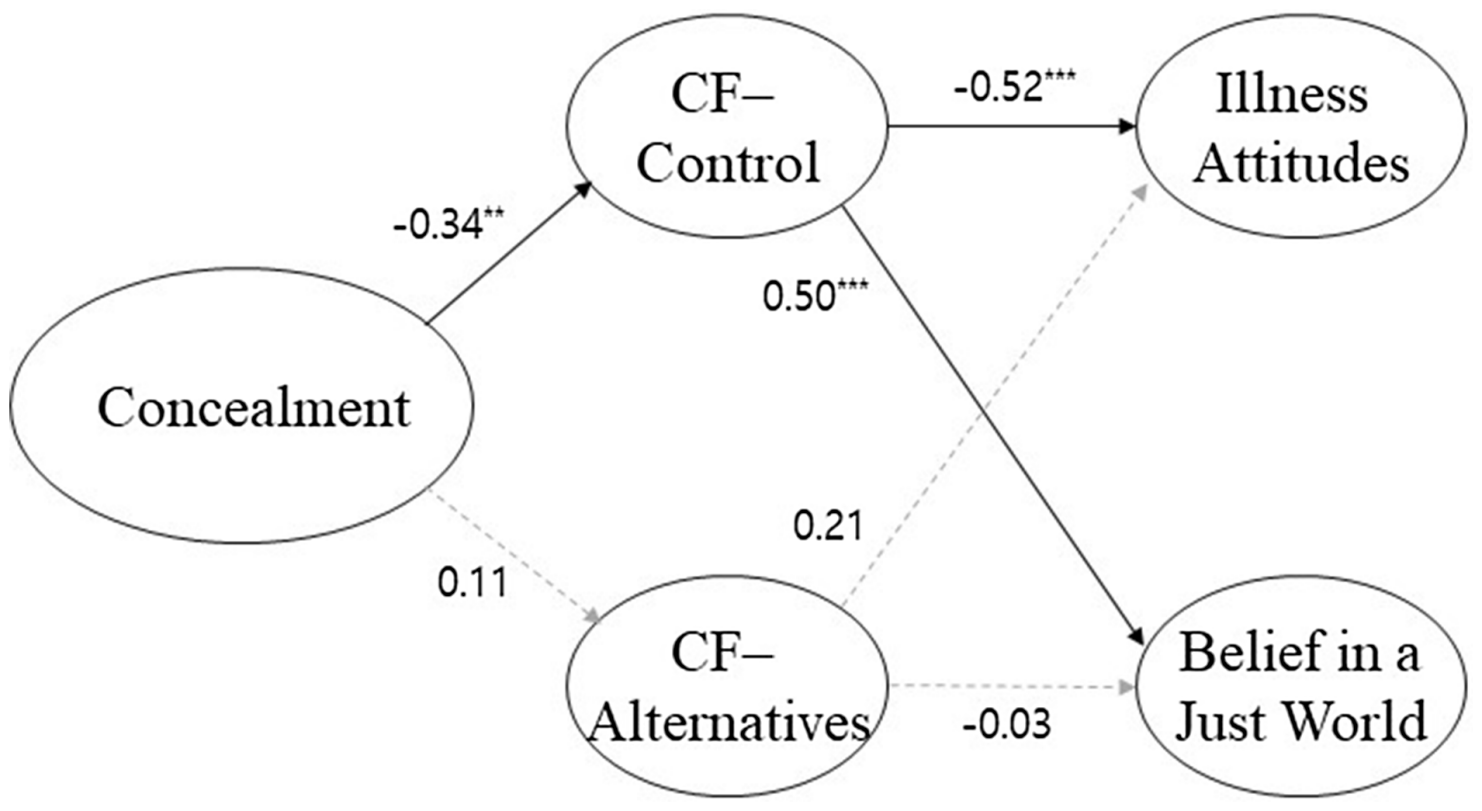
Verified Structural Model for Korean Subsample.

Figure 2 displays the final model’s path coefficients. Concealment tendencies were negatively related to control (*β* = −0.34, *р* < 0.001). Control was negatively related to illness attitudes (*β* = −0.52, *р* < 0.001) and positively related to belief in a just world (*β* = 0.50, *р* < 0.001).

In the modified model for the Chinese subsample, there is no path from concealment tendencies to belief in a just world, no path from control to belief in a just world, and no path from alternatives to illness attitudes. The chi-square value for the research model was 221.29, whereas that of the modified model was 227.45. When the degrees of freedom of the difference equaled 3, the chi-square value was 6.15, representing a significant change (*р* < 0.001). Therefore, we selected the modified model for this subsample, as well, due to its superior fit.

**Figure 3:**
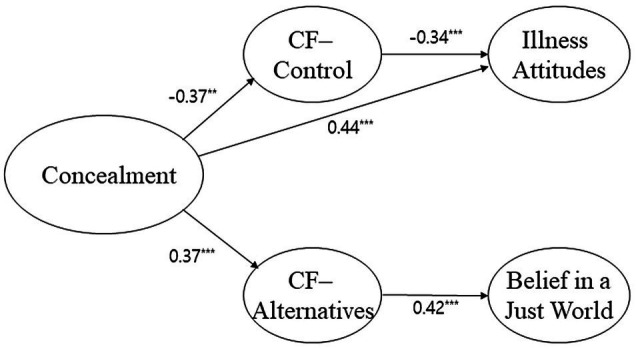
Verified Structural Model for Chinese Subsample.

Figure 3 displays the final model’s path coefficients. Every path coefficient was significant. Concealment tendencies were negatively related to control (*β* = −0.37, *р* < 0.001) but were positively related to alternatives (*β* = 0.37, *р* < 0.001) and to illness attitudes (*β* = 0.45, *р* < 0.001). Control was negatively related to illness attitudes (*β* = −0.34, *р* < 0.001). Alternatives was positively related to belief in a just world (*β* = 0.42, *р* < 0.001).

### Verification of Mediating Effects

For Korean participants, control fully mediated the relationship between concealment tendencies and illness attitudes. Additionally, control fully mediated the relationship between concealment tendencies and belief in a just world. Thus, to confirm the magnitude of control’s influence, we conducted a mediation effect verification using phantom variables (see Table 4). The mediating effect is considered significant if the confidence interval does not include zero. Control’s mediating effect size in the relationship between concealment tendencies and illness attitudes was 0.03, *p* < 0.01. The confidence interval’s lower and upper limits were 0.01 and 0.05, respectively, indicating a significant effect. Additionally, control’s mediating effect size in the relationship between concealment tendencies and belief in a just world was −0.03, *p* < 0.001. The confidence interval’s lower and upper limits were −0.07 and −0.01, respectively, indicating a significant effect.

**Table 4:** Verification results of mediating effect.

	Path	Mediating effect	95% CI	
			Lower	Upper
Korean subsample (*N* = 418)	CT → Control → IA	0.03^**^	0.01	0.05
	CT → Control → BJW	−0.03^**^	−0.07	−0.01
Chinese subsample (*N* = 400)	CT → Control → IA	0.07^***^	0.04	0.10
	CT → Alternatives → BJW	0.12^***^	0.07	0.19

*CT, concealment tendencies; IA, illness attitudes; BJW, belief in a just world; and CI, confidence interval*.

** *p < 0.01;*

*** *p < 0.001*.

For Chinese participants, control partially mediated the relationship between concealment tendencies and illness attitudes. However, alternatives fully mediated the relationship between concealment tendencies and belief in a just world. We used the same analysis outlined above to confirm the magnitudes of the respective influences of control and alternatives. We found that control’s mediating effect size in the relationship between concealment tendencies and illness attitudes was 0.07, *p* < 0.001. The confidence interval’s lower and upper limits were 0.04 and 0.10, respectively. Although this is a significant result, we confirmed that control partially mediated the relationship because of its significant direct effect on illness attitudes (*β* = 0.45, *р* < 0.001). Alternatives’ mediating effect size in the relationship between concealment tendencies and belief in a just world was 0.12, *p* < 0.001. The confidence interval’s lower and upper limits were 0.07 and 0.19, respectively; thus, this mediating effect was also significant.

## Discussion

We sought to examine the personally and socially maladaptive characteristics of concealment tendencies and to illuminate their process in a social atmosphere where anxiety caused by COVID-19 is prevalent. To this end, we examined both personal perceptions (illness attitudes) and social perceptions (belief in a just world) of those with strong concealment tendencies. Additionally, we examined the role of cognitive flexibility in the relationships among concealment tendencies, illness attitudes, and belief in a just world. Next, we discuss this study’s results and implications.

Concealment tendencies were negatively correlated with cognitive flexibility–control in both the Korean and Chinese subsamples. This indicates that individuals demonstrating higher levels of concealment tend also to have lower levels of cognitive flexibility with respect to control. Individuals with strong concealment tendencies are mindful that their shortcomings may be revealed through words or actions (Winnicott, 1960; Schlenker and Leary, 1982; Hewitt et al., 2003). However, because it is impossible to completely conceal their imperfections, they might experience anxiety (Clark, 2005). Moreover, greater obsession with concealing imperfections may lower their cognitive flexibility, which could lead to psychological maladjustment (Heo, 2011). In other words, people with a strong tendency to fear evaluation may be less cognitively flexible because they cannot easily accept others’ evaluations (Park and Hong, 2019). Thus, our results align with Shi and Qian’s (1998) finding that those who are easily embarrassed avoid, deny, and distort reality by concealing their problems rather than solving them in difficult situations.

Cognitive flexibility–control was significantly negatively correlated with illness attitudes in both subsamples. Higher levels of the control aspect of cognitive flexibility are associated with less negative attitudes toward disease. Cognitive flexibility increases adaptability and enables appropriate responses in difficult situations, which directly affects anxiety reduction mechanisms, including illness attitudes. Low cognitive flexibility, in contrast, increases the likelihood of experiencing depression and anxiety (Kim and Seo, 2009; Heo, 2011; Kim and Lee, 2013; Lee and Yang, 2015).

In all other relationships among the factors, the differences between the two countries were prominent. One notable difference between the results from the Korean and Chinese subsamples was the relationship between concealment tendencies and cognitive flexibility–alternatives. For Chinese, concealment tendencies were significantly positively correlated with cognitive flexibility–alternatives. Cognitive flexibility involves the ability to replace maladaptive thoughts with more adaptive ones, and the alternatives aspect refers to the ability to switch one’s cognitive framework in response to changing environmental stimuli (Wang et al., 2016). Therefore, we suggest that Chinese individuals, who often demonstrate strong concealment tendencies, are particularly adept at producing alternative explanations for events and solutions to problems.

Furthermore, for Chinese participants, cognitive flexibility–alternatives were significantly positively correlated with belief in a just world. That is, people with a stronger ability to devise alternative explanations and solutions are more likely to believe in a just world than their less cognitively flexible counterparts. This finding aligns with the results from Wang et al.’s (2019) study. The researchers found that cognitive flexibility influenced belief in a just world, with the alternatives aspect exerting greater influence than the control aspect.

Although belief in a just world was related to cognitive flexibility in both subsamples, the type of cognitive flexibility differed. For Koreans, the correlation with cognitive flexibility–control was significant while that with alternatives was not significant. For Chinese, the correlation with cognitive flexibility–control was not significant while that with alternatives was significant. We propose the two countries’ different social systems and values as a plausible explanation for these different results. Before discussing this further, we elaborate on the cross-cultural differences in the results.

We used structural equation modeling to verify the mediating effects of cognitive flexibility. We found that cognitive flexibility–control fully mediated the relationship between concealment tendencies and illness attitudes for Koreans and partially mediated this relationship for Chinese. Previous research with Koreans has shown that those with high levels of perfectionistic self-presentation, including concealment tendencies, tend to perform maladaptive cognitive-emotional control behaviors (Kang, 2020). People with strong concealment tendencies typically fear being rejected by others in interpersonal relationships (Burns, 1980; Weisinger and Lobsenz, 1981; Hewitt et al., 2003), and they tend to avoid acknowledging their mistakes. They even hide their emotions, which consequently makes them feel less control over their situation. Less control entails greater difficulty believing that the world is fair and more negative attitudes toward disease.

For Chinese participants (but not Korean participants), cognitive flexibility–alternatives fully mediated the relationship between concealment tendencies and belief in a just world. This supports a previous study’s finding that belief in a just world helps people reduce anxiety by coping with threats and restructuring justice at a cognitive level (Zhou, 2013).

Based on these results, it was apparent that although Korea and China have commonalities as collectivist cultures, there are cultural and psychological differences in the details. Regarding similarities, both Korea and China showed that individuals’ concealment tendencies were negatively related to the cognitive flexibility–control factor. They became negative about disease as their cognitive flexibility–control decreased. The cognitive flexibility–control factor relates to perceiving difficult situations as controllable (Dennis and Vander Wal, 2010). Both Korean and Chinese individuals in the collectivist culture are embarrassed when they misbehave in front of others and hide their behaviors (Lee and Kim, 2006; Wang, 2011). People willing to minimize their mistakes in public situations and hide their imperfect selves are more likely to become anxious and more sensitive to social pressure. This anxiety can eventually lead to having diseases related to stress (Lu et al., 2009). Therefore, concealment tendencies pursuing a perfect public image negatively influenced illness attitude in both subsamples.

Regarding the differences between the two countries’ results, we propose that the differences are due to divergent social systems, attitudes, and values. China has maintained a socialist government for decades. In socialist countries, the interests of the community tend to be prioritized over the interests of individuals. Something judged as being beneficial to the community is considered the most valuable. Correspondingly, if an individual or state has concealed a fact, the reason for concealment might be for the benefit of the community, and concealment might have been a deliberate choice. If people recognize the world in which these choices are made as orderly and stable, they may believe more strongly in a just world. This view of the social community is also revealed in the formation of public opinion (Ren and Xie, 2018). In 2016, a Communist Party of China (CPC) press conference suggested that public opinion must maintain the CPC’s principles and share the CPC’s thoughts, politics, and action. All forms of media must embody the CPC’s will, propagate its claims, defend its authority and unity, love and protect it, and advocate on its behalf (Ren and Xie, 2018). Chinese citizens generally accept the government-run media. One citizen states, “It is an inevitable process for the country to develop without losing its focus” (Kumendedalingnv1, 2013). Another frames the media situation thus: “It is not good to cause unnecessary social anxiety” (Dali858, 2010). Given these perceptions, we can partially understand why there are differences between countries in the relationships among concealment tendencies, alternatives, and belief in a just world.

Although concealment tendencies are associated with maladaptive traits in Korea, there are positive aspects in China. Because of the culture of the socialist system described above, personal concealment tendencies increase the cognitive flexibility–alternative factor, which positively affects the social attitude of belief in a just world. For example, Li et al. (2020) conducted a citizens’ survey in the first coronavirus outbreak area. They found that more than 85.7% of citizens were satisfied with the government’s countermeasures. Amid uncertainty or inequality, people tend to have a sense of stability by having hope and confidence (Zhen, 2013). For Chinese individuals, the stronger the concealment tendencies, the more they consider cognitive–alternative factors, reducing anxiety and protecting their positive self-images and belief in a just world. In summary, for Koreans, concealment tendencies are personal attitudes that could be maladaptive, whereas, for Chinese individuals, concealment tendencies have positive and negative effects.

This study’s results have several implications. First, we confirmed the relationships among concealment tendencies, illness attitudes, and belief in a just world. Our verification of mediating effects underscores the fact that cognitive factors influence personal attitudes and social viewpoints. Second, this study determined that people from two Asian countries exhibit differences in their concealment tendencies. Although most previous studies have focused on the negative effects of concealment, we suggest its potentially positive effect for Chinese individuals of increasing cognitive flexibility with respect to alternatives and strengthening belief in a just world. Third, our study results could prove useful in providing psychotherapy to those with illness anxiety. If clients with strong concealment tendencies demonstrate serious negative attitudes toward disease and resentment and criticism toward the world, their psychotherapists may need to explore clients’ cognitive flexibility. For Koreans, specifically, it would be more effective to address the control dimension instead of the alternatives dimension of cognitive flexibility. In contrast, for Chinese, it would be more effective to target the control dimension if treating individual attitudes (e.g., illness attitudes) and to target the alternatives dimension if treating social viewpoints (e.g., belief in a just world).

Our study also has several limitations. First, we collected data by sampling people from multiple regions of Korea and China, but generalizability may still be limited, as we did not sample from all regions. Future studies may benefit from expanded sampling procedures. Second, we utilized a self-report questionnaire, which reflects individual or situational factors when participants respond to the questionnaire. Furthermore, there is also the possibility of responding to the questionnaire based on social desirability. It is possible that participants’ true characteristics have been distorted in this regard. Future research could use more objective measures that consider the cultural backgrounds and values in each country as well as take a multifaceted approach (e.g., qualitative research). Finally, we used cross-sectional data measured by a self-report questionnaire for all variables. Therefore, there will be a limit to find a clear causal relationship.

## Data Availability Statement

The raw data supporting the conclusions of this article will be made available by the authors, without undue reservation.

## Ethics Statement

Ethical review and approval was not required for the study on human participants in accordance with the local legislation and institutional requirements. The patients/participants provided their written informed consent to participate in this study.

## Author Contributions

JH conceived and designed the analysis and contributed the data or analysis tools. KC and HJ collected the data. KC performed the analysis. KC, JH, and JJ wrote the paper. All authors read and approved the final manuscript.

## Conflict of Interest

The authors declare that the research was conducted in the absence of any commercial or financial relationships that could be construed as a potential conflict of interest.

## Publisher’s Note

All claims expressed in this article are solely those of the authors and do not necessarily represent those of their affiliated organizations, or those of the publisher, the editors and the reviewers. Any product that may be evaluated in this article, or claim that may be made by its manufacturer, is not guaranteed or endorsed by the publisher.
